# Integrated analysis of omics data using microRNA-target mRNA network and PPI network reveals regulation of Gnai1 function in the spinal cord of *Ews/Ewsr1* KO mice

**DOI:** 10.1186/s12920-016-0195-4

**Published:** 2016-08-12

**Authors:** Chai-Jin Lee, Hongryul Ahn, Sean Bong Lee, Jong-Yeon Shin, Woong-Yang Park, Jong-Il Kim, Junghee Lee, Hoon Ryu, Sun Kim

**Affiliations:** 1 Interdisciplinary Program in Bioinformatics, Seoul National University, Seoul, 151-747 Republic of Korea; 2 Department of Computer Science and Engineering, Seoul National University, Seoul, 151-744 Republic of Korea; 3 Bioinformatics Institute, Seoul National University, Seoul, 151-747 Republic of Korea; 4 Department of Pathology & Laboratory Medicine, Tulane University School of Medicine, New Orleans, LA 70112 USA; 5 Genome Medicine Institute and Department of Biochemistry, Seoul National University College of Medicine, Seoul, 110-799 Republic of Korea; 6 Samsung Genome Institute, Samsung Medical Center and Department of Health Sciences and Technology, Samsung Advanced Institute for Health Sciences and Technology, Sungkyunkwan University, Seoul, 135-710 Republic of Korea; 7 VA Boston Healthcare System, Boston, MA 02130 USA; 8 Boston University Alzheimer’s Disease Center and Department of Neurology, Boston University School of Medicine, Boston, MA 02118 USA; 9 Center for Neuromedicine, Brain Science Institute, Korea Institute of Science and Technology, Seoul, 136-791 Republic of Korea

**Keywords:** EWS, Ewsr1, Gnai1, MMIA, PPI, Network analysis

## Abstract

**Background:**

Multifunctional transcription factor (TF) gene *EWS/EWSR1* is involved in various cellular processes such as transcription regulation, noncoding RNA regulation, splicing regulation, genotoxic stress response, and cancer generation. Role of a TF gene can be effectively studied by measuring genome-wide gene expression, i.e., transcriptome, in an animal model of *Ews/Ewsr1* knockout (KO). However, when a TF gene has complex multi-functions, conventional approaches such as differentially expressed genes (DEGs) analysis are not successful to characterize the role of the *EWS* gene. In this regard, network-based analyses that consider associations among genes are the most promising approach.

**Methods:**

Networks are constructed and used to show associations among biological entities at various levels, thus different networks represent association at different levels. Taken together, in this paper, we report contributions on both computational and biological sides.

**Results:**

Contribution on the computational side is to develop a novel computational framework that combines miRNA-gene network and protein-protein interaction network information to characterize the multifunctional role of *EWS* gene. On the biological side, we report that EWS regulates G-protein, *Gnai1*, in the spinal cord of *Ews/Ewsr1* KO mice using the two biological network integrated analysis method. Neighbor proteins of *Gnai1*, G-protein complex subunits *Gnb1, Gnb2* and *Gnb4* were also down-regulated at their gene expression level. Interestingly, up-regulated genes, such as *Rgs1* and *Rgs19*, are linked to the inhibition of *Gnai1* activities. We further verified the altered expression of Gnai1 by qRT-PCR in *Ews/Ewsr1* KO mice.

**Conclusions:**

Our integrated analysis of miRNA-transcriptome network and PPI network combined with qRT-PCR verifies that Gnai1 function is impaired in the spinal cord of *Ews/Ewsr1* KO mice.

**Electronic supplementary material:**

The online version of this article (doi:10.1186/s12920-016-0195-4) contains supplementary material, which is available to authorized users.

## Background

Ewing sarcoma is the second most common bone and soft tissue tumor that predominantly afflicts children and adolescents [1–3]. Understanding biological mechanisms underlying this tumor is critical to the identification of new cancer therapy targets. The Ewing sarcoma gene (*EWS*)/EWS RNA-Binding Protein 1 (*EWSR1*), a transcription factor, encodes an RNA binding protein whose specific functional targets are still largely unknown [4]. In previous studies, fusion genes such as, *EWS-FLI-1, EWSR1-WT1, EWSR1-KLF17, EWSR1-ATF1,* and *EWSR1-CREB3L1*, are known to be produced by rearrangement of the *EWSR1* gene with different gene fusion partners and these fusion genes have functions related to a variety of soft tissue tumors [5–9]. To characterize functions of *EWS*, we used RNA-seq gene expression data and miRNA expression data measured by using the spinal cord samples of *Ews/Ewsr1* knock-out (KO) mouse and wild type.

### Motivation

Multi-function genes interact with a number of coding and non-coding genes and perform a variety of functions depending on cell conditions and tissue types. Multi-function gene *EWSR1* is known to regulate *Drosha* and microRNAs that inhibits RNA splicing [10, 11]. However, it is still unknown which genes are regulated by and which biological functions are related to *EWSR1*. To characterize functions of *EWSR1*, we used a well-known differentially expressed gene (DEG) set analysis. We performed functional analysis of top 200 up-regulated DEGs and top 200 down-regulated DEGs (2 % of the whole genes) using gene ontology (GO) and KEGG pathway. From the GO analysis, we found 322 genes of 400 top DEGs were involved in 44 GO terms in the GOTERM_BP_FAT category which is the summarized version of Biological Processes in the Gene Ontology (Additional file 1A). Top three GO terms with the largest number of genes were ion transport, immune response, and homeostatic process. It is not clear how these three biological processes are related to *EWS*. In addition, we tried molecular function GO terms, which did not produce coherent biological functions related to *EWS*. From the KEGG pathway result, 93 of 400 genes hit 140 pathways. Only two pathways had more than 10 genes: metabolic pathway and cell adhesion molecules. Most of the pathways were not significant. Overall, GO and KEGG pathway analysis using DEGs did not produce meaningful clues on the role of *EWS*.

For the analysis of miRNA expression data, it is not clear how to perform an integrated analysis of gene expression data and miRNA expression data. In addition, a multifunction gene can play roles at various levels such as transcription, gene regulation, translation and protein activity level. To address this computational challenge, we developed a novel computational framework for the characterization of *EWS* multifunctional gene using gene expression data and miRNA expression data measured under a knockout condition of the multifunctional gene. The framework utilized microRNA-target gene network and Protein-Protein interaction (PPI) network and incorporates the two networks in a workflow. The workflow of the framework can be viewed as an effort to model the role of *EWS* at various levels, DEG analysis at the transcription level, the microRNA-target gene network analysis at the gene regulation level, and PPI network analysis at the translation and protein activity level.

## Methods

**Fig. 1:**
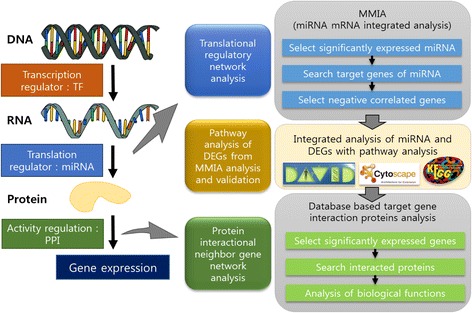
Illustration of the workflow of the pipeline. Transcription factor (TF) gene has multiple functions to regulate transcription. Generated mRNAs are regulated by microRNA and translated proteins have functions with interacted proteins and molecules. RNA sequencing data and microRNA (miRNA) microarray data are generated from spinal cord extraction in *Ews/Ewsr1* knockout and wild type mice. SAM (Significance Analysis of Microarrays) is used for selection of significantly expressed miRNA from miRNA microarrays. TargetScan and miRDB were used to predict target genes of miRNAs. From RNA sequencing data, gene expression values are mapped to the reference genome data using Tophat. Then negative correlated differentially expressed genes (DEGs) are selected. Significantly expressed microRNA target genes have many interacting proteins. Specific target gene interactional neighbor proteins are searched in the STRING DB. PPI network analyzed with gene expression value. Analysis results of miRNA-mRNA network and PPI network are integrated by analyzing correlation in expression levels. Regulated genes further are analyzed and visualized with DAVID, KEGG and Cytoscape

We developed a three-step pipeline for the integrated analysis of omics data using mRNA-microRNA network and protein-protein interaction network. We describe the workflow and computational methods used in each step in this section. Figure 1 illustrates the workflow of the proposed omics data analysis pipeline. In “Results” section, we discuss output from each step in detail.

### Step 1. MicroRNA-target gene regulation network analysis

- Input: gene expression data, miRNA expression data
- Output: differentially expressed miRNAs and their target genes

To investigate roles of *EWS*, we analyzed the translational regulatory network. The microRNA-target gene integrated network analysis was performed following the strategy in MMIA [12].

#### Selection significantly expressed microRNAs

We selected significantly up- or down-regulated microRNAs in the *Ews/Ewsr1* KO condition compared to the wild type condition. To select significantly differentially expressed miRNAs from microarray data, we used the SAM (significance analysis of microarrays) tool package [13] (More information in the detailed method section).

#### Prediction of microRNAs target genes

After selecting significantly expressed microRNAs, we predicted regulatory target genes of the selected differentially expressed microRNA by TargetScan [14] and miRDB [15, 16].

#### Reselection target genes by correlation

We further investigated miRNA and gene target relationship by measuring negative correlation in expression levels between miRNAs and genes targeted by miRNAs since up-regulated microRNA inhibits translation of mRNA.

### Step 2. Pathway analysis of DEGs from MMIA analysis and validation

- Input: DEGs selected in Step 1
- Output: important pathways related to EWS and key genes in the pathways

#### Differentially Expressed Gene (DEG) analysis

Differentially expressed genes (DEGs) analysis of NGS RNA-seq was performed in the following steps. First, adaptor sequences of reads in raw data were trimmed. The Ensembl mouse reference genome sequence was downloaded for mapping short reads. Bowtie [17] was used to build an index of the reference genome sequence for alignment. Trimmed reads were then mapped to the reference genome sequence using Tophat2 [18]. Finally, Cufflinks was used to calculate gene expression levels. We compared gene expression values and selected DEGs by using Cuffdiff in the Cufflinks package [19].

#### Integrated analysis of miRNA and mRNA expression data

15 differentially expressed miRNAs were found to target 4342 genes based on TargetScan and miRDB. To further screen target genes, we integrated miRNAs target information and mRNA-seq based gene expression levels. The negative correlation analysis reduced the number of targets to 1338 genes. The negative correlation analysis is based on the techniques in [20, 21]. The rationale for the negative correlation analysis is that if a miRNA targets a gene the expression levels of the miRNA and the gene should have negative correlation due to the regulatory effect of miRNA on the target gene. These DEGs were then analyzed by GSEA (Gene Set Enrichment Analysis) using DAVID (The Database for Annotation, Visualization and Integrated Discovery) [22].

#### Pathway analysis

To characterize functions of selected target DEGs by negative correlation in the spinal cord of *Ews/Ewsr1* KO mice, we performed biological pathway analysis using the KEGG mapper [23]. KEGG mapper highlighted DEGs with colors: up-regulated DEGs as red, down-regulated DEGs as blue, and other DEGs as light green. In addition, we performed additional pathway interpretation based on gene ontology by using ClueGO [24], a Cytoscape [25] plug-in, that analyzes biological pathway interpretation with KEGG ontology (2014 latest version) to integrate Gene Ontology (GO) terms and KEGG/BioCarta pathways to generate a functionally organized GO/pathway term network.

#### Verification of Gnai1 expression by Quantitative real-time PCR (qRT-PCR)

To verify whether the expression of target genes is correlated with the analysis, we performed qRT-PCR using RNA isolated from the spinal cords of *Ews/Ewsr1* WT and KO mice.

### Step 3. Protein-protein interaction network analysis

- Input: Key genes identified in Step 2
- Output: G protein complex genes and regulators

After selecting the key gene in Step 2, we investigated the biological functions of the genes by extending gene sets with neighboring genes of the key gene.

#### Selection significantly expressed gene

From gene set analysis (GSA) and pathway analysis (see the detailed methods section), we selected specific genes.

#### Search for proteins that interact with the selected gene

Protein-protein interaction (PPI) analysis of genes neighboring the key gene was performed by using STRING (Search Tool for the Retrieval of Interacting Genes/Proteins) [26], the most widely used database of known and predicted protein interactions.

#### Analysis of biological functions

Relationship between the key gene and neighbor genes was investigated by performing the literature search. When we considered the relationship among genes, we also considered the regulatory roles of genes, i.e., activators or repressors, if applicable. For the regulatory relationship, we considered gene expression change information.

## Results

### Analysis of multifunctional *EWS* by using the network-based workflow

In this section, we present the result from each computational step of the workflow (Fig. 1).

### Step 1. Translational regulatory network analysis: MicroRNA-mRNA network

#### Selection of differentially expressed miRNAs

We selected 18 significantly expressed miRNAs from the total 1193 mouse miRNAs by SAM tool. 15 miRNAs expression level were significantly up-regulated, and 3 miRNAs were down-regulated in the *Ews/Ewsr1* KO mice against WT mice (Additional file 2). In the order of the significance score by SAM, 15 up-regulated miRNAs are *mmu-miR-127, mmu-miR-410, mmu-miR-433, mmu-miR-138, mmu-miR-181c, mmu-miR-382, mmu-miR-19b, mmu-miR-381, mmu-miR-666-3p, mmu-miR-376a, mmu-miR-873, mmu-miR-181a, mmu-miR-383, mmu-miR-181b,* and *mmu-miR-99b*. Down-regulated 3 miRNAs were *mmu-miR-1224, mmu-miR-9-3p*, and *mmu-miR-26a* in the order of the significance score by SAM. Analysis of potential biological functions of these miRNAs was performed by using genes targeted by the miRNAs (see the DEG analysis from RNA-seq data result section).

#### Prediction of target mRNA regulated by selected miRNA

To perform the integrated analysis of miRNA and their target genes, we need to predict targets of miRNAs. Predicted target genes of miRNAs were collected by using TargetScan and miRDB. 5,779 and 5,448 genes were predicted by TargetScan and miRDB, respectively. 1,927 genes were targeted by multiple miRNAs in the prediction result of TargetScan, and 2,371 genes were multiply targeted according to miRDB. After discarding repeatedly predicted genes, a total of 4,342 genes were predicted as targets of 15 differentially expressed miRNAs. Only 36 % (1,587 genes) of predicted target genes were predicted by both TargetScan and miRDB. In other words, the genes targeted by each miRNAs of prediction results by TargetScan and miRDB do not agree much (Additional file 3). 4,342 target genes predicted by both TargetScan and miRDB were further analyzed by performing a negative correlation analysis to sort out potentially true miRNA-gene relationships (see the next section).

#### Negative correlation analysis of DEGs with DE microRNA

**Fig. 2:**
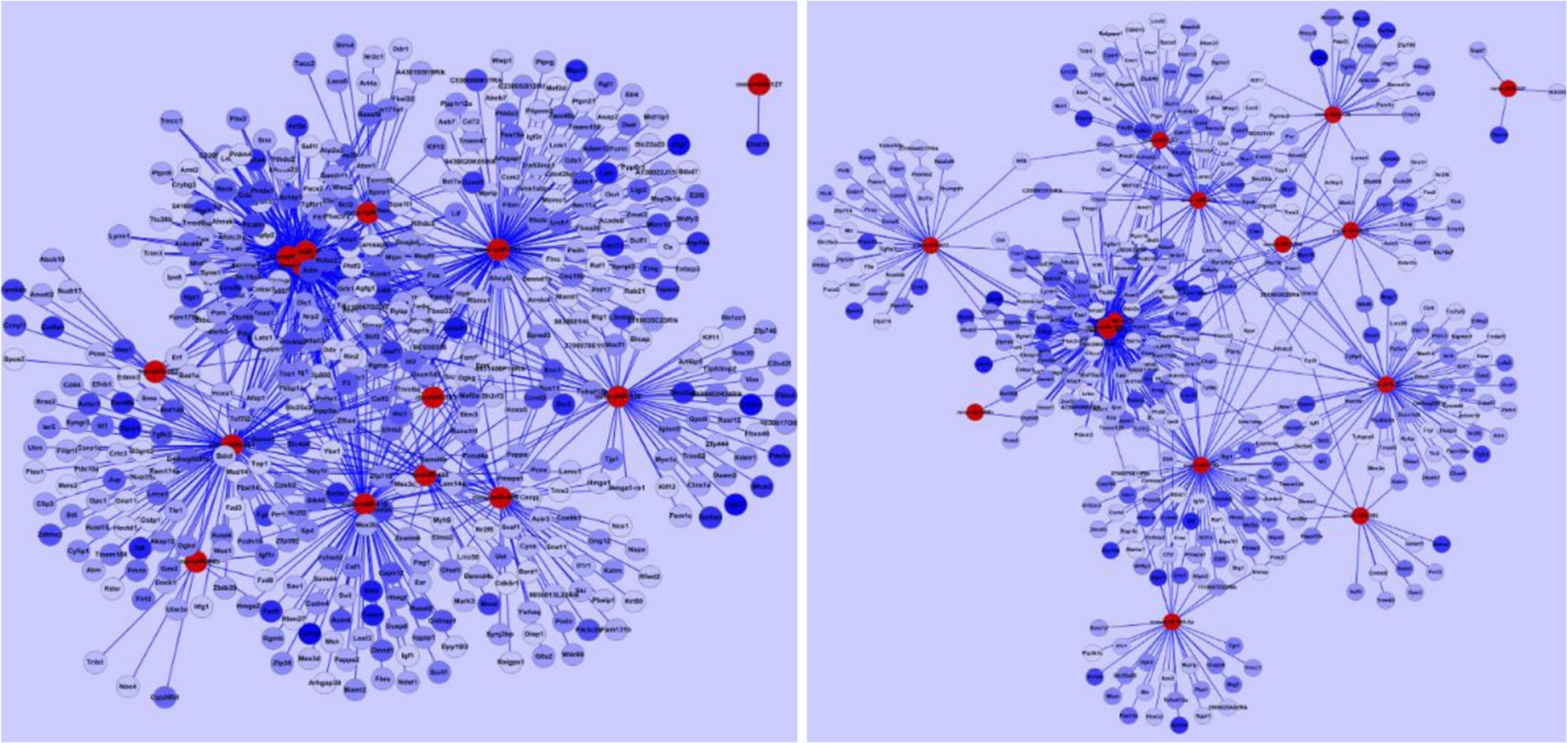
Network of microRNAs and mRNAs. Up-regulated miRNAs (Red nodes) are selected by SAM. Target genes (mRNAs, blue nodes) of selected miRNAs are predicted by TargetScan (left) and miRDB (right). Down-regulated genes targeted by up-regulated miRNA are selected from each predicted results. miRNA-mRNA interaction network is drawn by Cytoscape. Color intensity denotes the level of gene expression

Predicted target genes were further screened by considering negative correlations in expression levels between miRNA and each of its target genes. The rationale for the negative correlation analysis is that miRNA degrades its target genes, thus a higher expression level of miRNA should result in a lower expression level of its target. We applied the same technique used in [14, 15]. Negatively correlated miRNA-mRNA interaction network of miRNAs and their target DEGs were visualized by using Cytoscape (Fig. 2). In Fig. 2, significantly up-regulated 15 miRNAs are in red color, and negative correlated target DEGs are in blue color. Color intensity denoted the level of gene expression. As a result of the correlation analysis, 4,342 genes were reduced to 860 genes. Among the 860 DEGs, 339 target genes were targeted by multiple miRNAs.

### Step 2. Pathway analysis of DEGs from MMIA analysis and validation

#### KEGG pathway analysis of DEGs gene set targeted by miRNA

We mapped the 860 negatively correlated DEGs to the KEGG pathway using the KEGG mapper. 201 pathways were hit by the negatively correlated DEGs. We selected 13 pathways with eight or more gene hits. Metabolic pathways, calcium signaling pathway, PI3K-Akt signaling pathway, axon guidance, pathways in cancer, MAPK signaling pathway, tight junction, dilated cardiomyopathy, circadian entrainment, proteoglycans in cancer, regulation of actin cytoskeleton, cholinergic synapse and focal adhesion pathways were selected. Analysis of KEGG pathways of DEGs were highlighted in colors chosen by KEGG mapper. Blue color genes were down-regulated genes, and red color genes were up-regulated genes in the pathways of *Ews/Ewsr1* KO mice (Additional file 4). Color intensity denoted the level of gene expression.

#### Gene ontology based network analysis

**Fig. 3:**
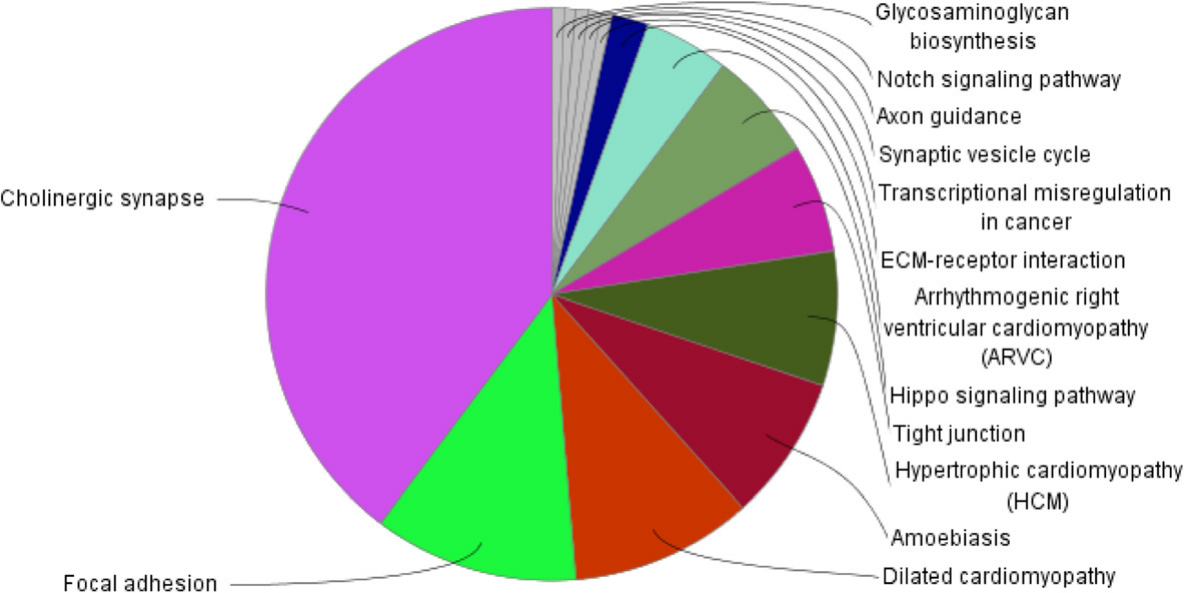
Venn diagram generated by ClueGO. ClueGO analyzes KEGG ontology of selected down-regulated genes which are targeted by up-regulated miRNA. Cholinergic synapse pathway is showed highly clustered by down-regulated gene pathways

Networks of negatively correlated target DEGs in terms of KEGG ontology were generated using ClueGO (Fig. 3). “Cholinergic synapse pathway” term was highly clustered by down-regulated DEGs belonging pathways. ECM-receptor interaction pathway, focal adhesion pathway, tight junction pathway, and action cytoskeleton regulation pathway were mostly correlated with selected down-regulated DEGs. *Gnai1*, which is most significantly down-regulated in the cholinergic synapse pathway, was selected for further investigation. More discussion on biological functions of these pathways is presented in the Conclusion section.

#### qRT-PCR of *Gnai1*

**Fig. 4:**
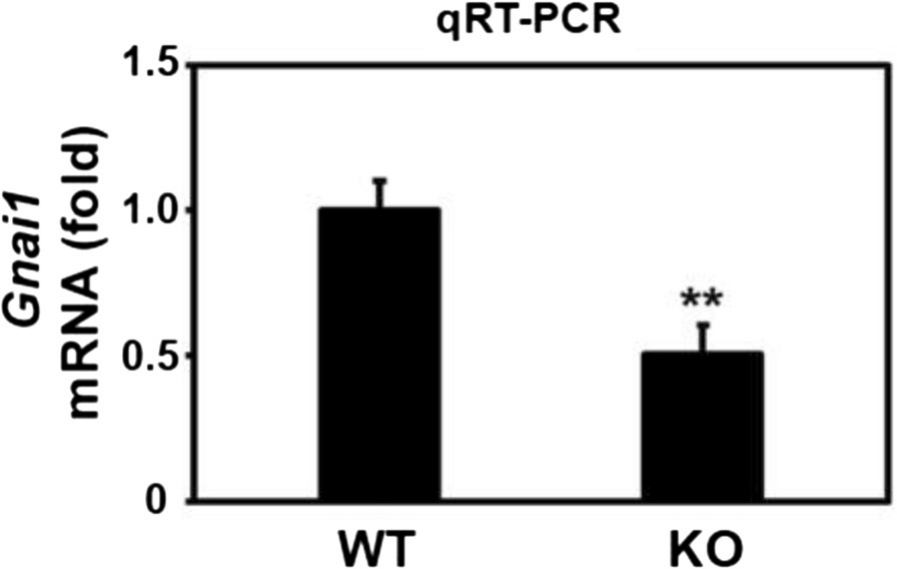
Verification of altered *Gnai1* expression in *Ews/Ewsr1* WT and KO mice. The gene expression level of Gnai1 was significantly lower in the spinal cords of *Ews/Ewsr1* KO mice (*n* = 6) compared to EWS WT mice (*n* = 6). The bar graph represents average ± standard error mean (SEM). **, Significantly different at *p* < 0.01 by Student T-test

qRT-PCR was performed to confirm the difference of *Gnai1* expression in the spinal cords of *Ews/Ewsr1* WT and KO mice. Average gene expression levels of *Gnai1* in *Ews/Ewsr1* KO mice were significantly lower than those in *Ews/Ewsr1* WT mice. This data validated that Gnai1 expression level was down regulated in *Ews/Ewsr1* KO mice (Fig. 4).

### Step 3. Protein-protein interactions network analysis

**Fig. 5:**
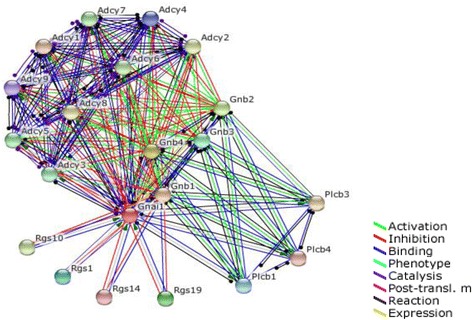
PPI network of Gnai1 from the STRING DB

**Fig. 6:**
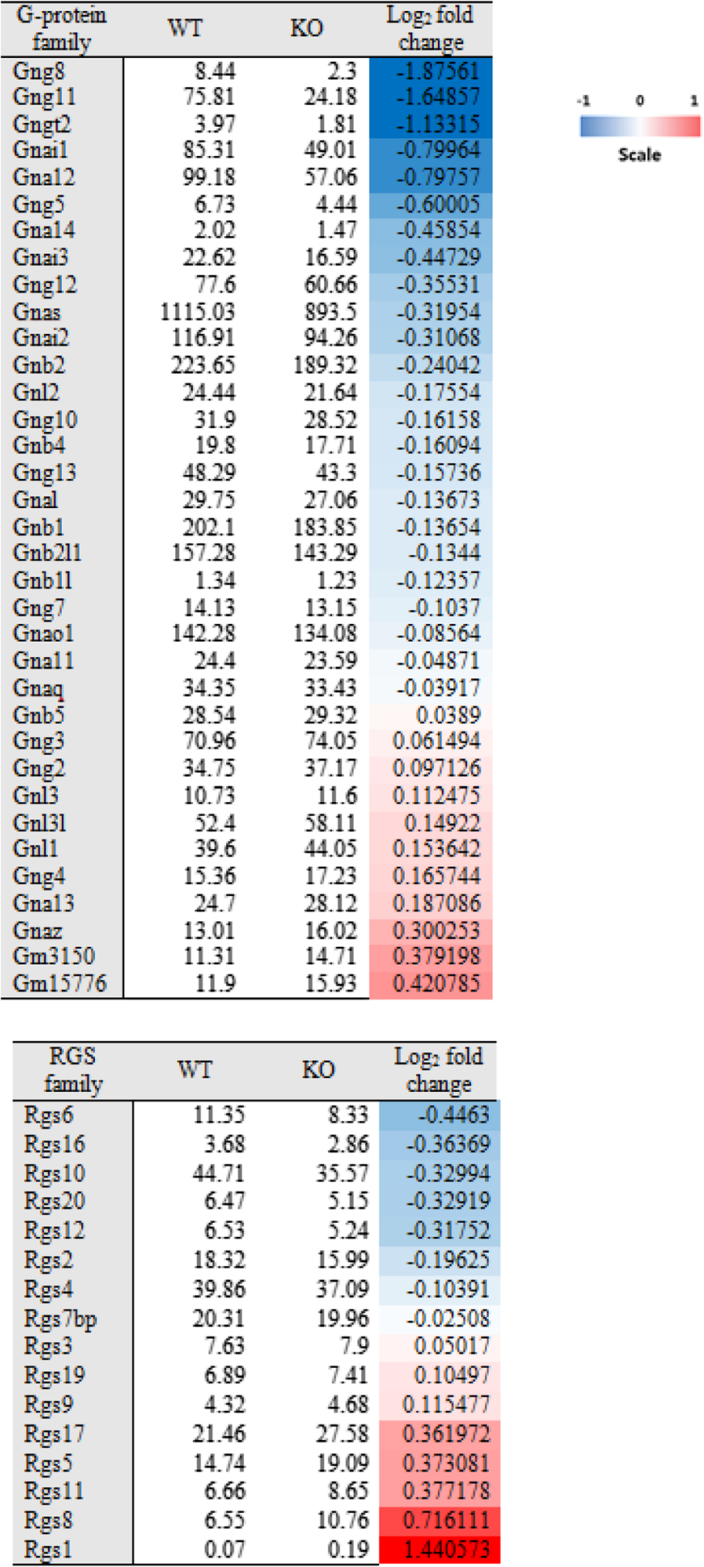
G-proteins and RGS (regulator of G-protein) expression level and log2 fold change value in *Ews/Ewsr1* wild type and knock-out

We selected Gnai1 that is down-regulated in cholinergic synapse pathways and action cytoskeleton regulation pathway. To investigate the effect of down-regulation of *Gnai1*, we used the STRING protein-protein interaction network DB. In the PPI network, genes neighboring *Gnai1* were further investigated for their biological functions. Looking at gene expression values, we were able to confirm the relationship between G-protein genes and *RGS* genes. Genes neighboring *Gnai1* were selected by using STRING (Fig. 5). Top 20 interacted genes are shown in Table 1. *Gnai1* and G-protein related genes, such as *Gnb1, Gnb2* and *Gnb4*, were down-regulated at their gene expression level (Fig. 6). In contrast, *Rgs1* and *Rgs19*, regulators of G-protein signaling genes that are associated with the inhibition of Gnai1 function, were up-regulated (Fig. 6).

**Table 1:** Top 20 interacted genes with Gnai1 from the STRING DB

Gene Symbol	Prediction Score	Binding	Inhibition
*Gnb1*	0.994	Yes	
*Gnb4*	0.98	Yes	
*Gnb2*	0.98	Yes	
*Rgs19*	0.979	Yes	Yes
*Gnb3*	0.978	Yes	
*Rgs1*	0.976	Yes	Yes
*Plcb1*	0.974	Yes	
*Adcy4*	0.973	Yes	Yes
*Adcy9*	0.973	Yes	Yes
*Rgs14*	0.972	Yes	Yes
*Plcb4*	0.97	Yes	
*Adcy1*	0.97	Yes	Yes
*Plcb3*	0.97	Yes	
*Adcy8*	0.969	Yes	Yes
*Adcy2*	0.969	Yes	Yes
*Rgs10*	0.969	Yes	Yes
*Adcy6*	0.967	Yes	Yes
*Adcy7*	0.967	Yes	Yes
*Adcy5*	0.966	Yes	Yes
*Adcy3*	0.966	Yes	Yes

These gene are sorted by prediction score. 13 genes are related to inhibition with Gnai1

## Discussion

### Potential interaction map of *EWS*, *RGS*, and G-protein complex genes

**Fig. 7:**
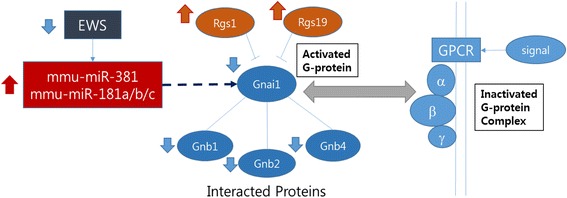
Roles of G proteins and its regulatory mechanisms by miRNAs in the spinal cord of *Ews/Ewsr1* KO mouse. Direction of arrow means with a change of gene expression level in *Ews/Ewsr1* KO mice. Upper arrows are up-regulated gene expression level, and bottom arrows are the opposite

A growing body of evidence shows multifunctional roles of the *EWS/EWSR1* fusion oncoproteins [5, 7–9]. However, the role of wild-type (WT) *EWS/EWSR1* is not fully understood yet. *EWS/EWSR1* deficiency contributes to the failure of precursor B lymphocyte development and leads to the premature cellular senescence in mouse embryonic fibroblasts (MEFs) [27, 28]. It seems likely that the WT *EWS/EWSR1* protein exhibits many different cellular functions in a cell-type specific manner. In the spinal cord of *Ews/Ewsr1* KO mice, microRNAs, such as *mmu-miR-381* and *mmu-miR-181a/b/c* were up-regulated. These microRNAs suppressed expression of *Gnai1* (Gi Protein Alpha subunit). Concurrently, *RGS* (Regulator of G-protein Signaling) genes, *Rgs1* and *Rgs19*, were up-regulated, which repressed *Gnai1* activity. In addition, G Protein Beta subunit genes, *Gnb1, Gnb2* and *Gnb4* were down-regulated. Thus in the *Ews/Ewsr1* KO condition, G protein complex was not formed (Fig. 7).

Since *Gnai1* was down-regulated, it is proposed that Gnai1 may be unable to inhibit downstream adenylate cyclase genes, such as *Adcy9* and *Adcy4*, in cholinergic synapse pathway. Adenylate cyclase catalyzes the conversion of ATP to cAMP, and the cAMP regulates cAMP-proteins, transcription factors, and cAMP-dependent kinases. Adenylate cyclase is an enzyme with key regulatory roles, and Adenylate cyclase regulator Gnai1 has important roles in cholinergic synapse.

Our study presents for the first time that *Ews/Ewsr1* deficiency modulates microRNA processing in the spinal cord. Notably, increased levels of *mmu-miR-381* and *mmu-miR-181a/b/c* were directly associated with the down regulation of G protein complex in the spinal cord of *Ews/Ewsr1* KO mice. We have previously shown that *Ews/Ewsr1* deficiency leads to abnormal microRNA processing and skin development via Drosha-dependent pathway [10]. Furthermore, we found that *Ews/Ewsr1* deficiency reduces the expression of Uvrag (UV radiation resistance associated) gene at the post-transcription level via *mmu-miR-125a* and *mmu-miR-351* [29]. Interestingly, the reduction of Uvrag by *mmu-miR-125a* and *mmu-miR-351* impaired autophagy function in *Ewsr1* knockout (KO) MEFs and KO mice. Considering that G protein-coupled signaling transduction pathway is very complex, the Gnai1-dependent cellular function and mechanism in in vitro and in vivo models of *EWSR1* deficiency remains to be determined in future studies.

## Conclusion

We developed a computational framework for the analysis of the multifunction TF *EWS* gene and showed that *EWS* has a significant role in the regulation of G protein complex. Since a multifunction TF gene has a complicated biological functions at various levels, such as transcription, gene regulation, and protein levels, powerful analysis tools are needed. Our method utilized miRNA-target gene network and protein-protein interaction network and combined multiple tools in a single computational framework.

We analyzed the miRNAs and mRNA data in the spinal cord of *Ews/Ewsr1* KO mice, and selected all significantly differentially expressed miRNAs and negative correlated DEGs. We constructed an interaction network with selected miRNAs and mRNAs and analyzed the GSEA and related pathways. From the result of pathway analysis, we identified significantly down-regulated *Gnai1* gene in the cholinergic synapse pathway that is highly clustered by down-regulated DEGs belonging pathways. *Gnai1* was verified by qRT-PCR, and analyzed about PPI sub-networks. *Gnai1* was suppressed by *mmu-miR-381* and *mmu-miR-181a/b/c*, and inhibited by *Rgs1* and *Rgs19* in the spinal cord of *Ews/Ewsr1* KO mice. As a future work, we plan to develop a software package for the analysis of multifunction TF genes.

### Material & detailed methods

#### NGS data

RNA sequencing data and microRNA microarray data those were generated from the spinal cord tissue samples of *Ews/Ewsr1* WT and KO mice [10].

#### Differentially expressed miRNA analysis

Differentially expressed miRNAs were selected from miRNA microarray data by using the samr (SAM: Significance Analysis of Microarrays, version: 2.0) package in Bioconductor. We used “two-class unpaired” option with 1000 permutations. SAM generated an interactive plot of the observed vs. expected (based on the permuted data) d-values. The user can dynamically change thresholds for significance to set the value of the tuning parameter delta. We set the delta to 2 to reduce the numbers of selected significant miRNAs.

#### MicroRNA target Gene prediction

We collected target genes of differentially expressed miRNAs using TargetScan and miRDB. TargetScan predicts biological targets of selected miRNAs by searching for the presence of conserved 8mer and 7mer sites that match the seed region of each miRNA. miRDB is a database of predicted miRNA targets in animals. MicroRNA targets in miRDB were predicted by using SVM (support vector machine) based prediction program. Only 22 % of predicted target genes by TargetScan and miRDB agreed. Since we were unable to decide which predicted gene are correct and we used all predicted target genes.

#### Reference genome sequence for alignment

We downloaded and used Ensembl reference genome sequence (Mus_musculus.GRCm38.70) for reads mapping [30].

#### GTF (General Transfer Format) file for gene annotation

After the alignment, we calculated the FPKM (fragment per kb exon model) values of each gene by Cufflinks with Ensembl gene model (Mus_musculus.GRCm38.70) [31].

#### Preprocessing of RNA-sequence data for DEG analysis

Before mapping reads, we clipped two adaptor sequences of paired-end RNA-seq data. For trimming, we allowed 2 mismatch of adaptor sequences to short reads. After the trimming process, we discarded reads of 18 bp or shorter.

Used trimming processing adaptor sequences show the next lines.

READ1 adaptor sequence: GATCGGAAGAGCACACGTCTGAACTCCAGTCAC

READ2 adaptor sequence: AGATCGGAAGAGCGTCGTGTAGGGAAAGAGTGTAGATCTCGGTGGTCGCCGTATCATT

#### DEG (differently expressed gene) analysis from RNA-seq NGS data

Paired-end total RNA-sequencing raw data were generated by Illumina HiSeq 2000. Each of the numbers of reads in raw data of wild type and *Ews* Knockout 3-week-old mice spinal cord samples show Table 2. After adaptor sequence trimming process for discarding of low quality sequence, the number of trimmed reads for each samples show Table 2. These amount of reads is sufficient for DEG analysis. After reference genome indexing, trimmed short reads were mapped to the reference genome by Tophat. The ratios of mapped reads for each samples were 81.72 and 81.5 %. The mapping ratios were higher than 80 % for all samples and variations in the mapping ratio across the samples were very small. Thus we believe that results of analysis for RNA sequencing experiment and short read processing were satisfactory. We quantified the expression level of each gene using Cufflinks based on the gene information from Ensembl.

**Table 2:** Number of reads and ratios of mapped reads in the process of RNA-seq analysis

Samples	Number of reads in raw data	Number of reads after trimming	Ratios of mapped read
Wild type 3-week-old sample	37,804,437	37,138,795	81.72 %
*Ews* Knockout 3-week-old sample	40,139,625	39,501,475	81.50 %

The numbers of reads in RNA-seq raw data of wild type and *Ews* Knockout 3-week-old mice spinal cord samples were 37,804,437 and 40,139,625. The number of trimmed reads after adaptor sequence trimming process for each samples were 37,138,795 and 39,501,475. The ratios of mapped reads for each samples were 81.72 and 81.5 %

#### Quantitative real-time PCR

Total RNA was extracted from the spinal cord of *Ews/Ewsr1* WT and KO mice by TRIzol reagent (MRC, Cincinnati, OH, USA) as previously described [10]. RNA was measured in a spectrophotometer at 260-nm absorbance. RNA analysis was conducted as follows. Fifty nanograms of RNA were used as a template for qRT-PCR amplification, using SYBR Green Real-time PCR Master Mix (Toyobo, Osaka, Japan). Primers were standardized in the linear range of cycle before the onset of the plateau. Mouse GAPDH was used as an internal control. Two-step PCR thermal cycling for DNA amplification and real-time data acquisition were performed with an ABI StepOnePlus Real-Time PCR System using the following cycle conditions: 95 °C for 1 min × 1 cycle, and 95 °C for 15 s, followed by 60 °C for 1 min × 40 cycles. Fluorescence data were analyzed by the ABI StepOnePlus software and expressed as, Ct, the number of cycles needed to generate a fluorescent signal above a predefined threshold. The ABI StepOnePlus software set baseline and threshold values.

## Abbreviations

DAVID, the database for annotation, visualization and integrated discovery; DEG, differentially expressed gene; DNA, deoxyribonucleic acid; EWS, Ewing’s Sarcoma; EWSR1, EWS RNA-binding protein 1; FPKM, fragments per kilobase of exon per million fragments mapped; Gnai1, Gi protein alpha subunit; GO, gene ontology; GSEA, gene set enrichment analysis; KEGG, Kyoto encyclopedia of genes and genomes; KO, knock-out; MMIA, microRNA and mRNA integrated analysis; PPI, protein-protein interaction; qRT-PCR, quantitative real-time PCR; RGS, regulator of G-protein signaling; RNA, ribonucleic acid; RNA-seq, whole transcriptome sequencing; STRING, Search Tool for the Retrieval of Interacting Genes/Proteins; TF, Transcription factor

## Additional files

Additional file 1: Table S1. Top 400 DEGs analysis result. A) GO term result of DAVID analysis. B) KEGG pathway result list. (XLSX 19 kb) Additional file 2: Figure S1. Graphic plotting of miRNA microarray analysis by SAM. Red dots are significantly up-regulated miRNAs and green dots are down-regulated. In the table of SAM result, columns are score, numerator, denominator, fold change and q-value. (DOCX 34 kb) Additional file 3: Table S2. The number of genes targeted by each miRNAs by using TargetScan and miRDB. Prediction results by TargetScan and miRDB do not agree much. Union of target genes were further analyzed by performing. (DOCX 15 kb) Additional file 4: Figure S2. The cholinergic synapse pathway related with significantly down-regulated genes by ClueGO. Selected DEGs are highlighted in colors chosen by KEGG mapper. Blue genes are down-regulated genes, and red genes are up-regulated genes in *Ews/Ewsr1* KO mice compared to WT mice. Green color genes are not changed. (DOCX 53 kb)
