# Supplementary material for: Integrated analysis of omics data using microRNA-target mRNA network and PPI network reveals regulation of Gnai1 function in the spinal cord of Ews/Ewsr1 KO mice
Source: BMC Med Genomics. 2016 Aug 12;9(Suppl 1):33. doi: 10.1186/s12920-016-0195-4 (PMC4989891; doi:10.1186/s12920-016-0195-4)
Supplement: Additional file 2: Figure S1. — Graphic plotting of miRNA microarray analysis by SAM. Red dots are significantly up-regulated miRNAs and green dots are down-regulated. In the table of SAM result, columns are score, numerator, denominator, fold change and q-value. (DOCX 34 kb) [file 12920_2016_195_MOESM2_ESM.docx]

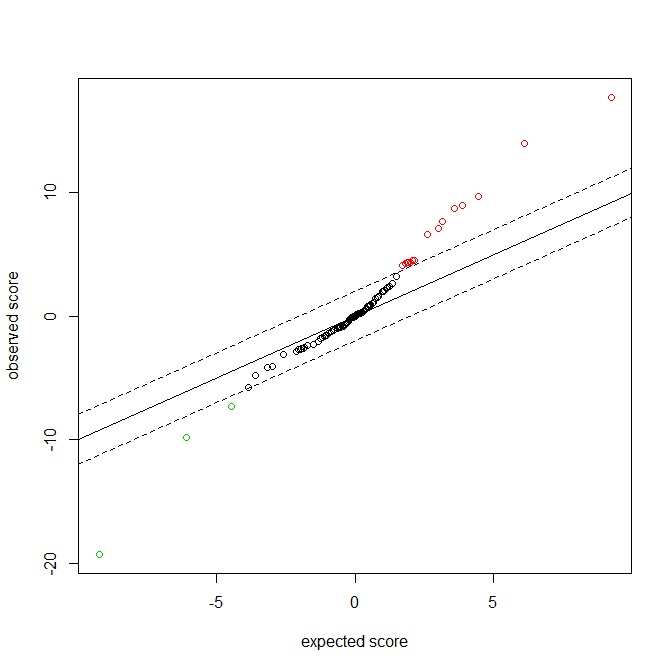


| **Gene ID** | **Score** | **Numerator** | **Denominator** | **Fold Change** | **q-value (%)** |
| --- | --- | --- | --- | --- | --- |
| mmu-miR-127 | 17.75175304 | 0.20740989 | 0.01168391 | 1.154613414 | 0 |
| mmu-miR-410 | 13.97305565 | 0.36733484 | 0.026288798 | 1.289967612 | 0 |
| mmu-miR-433 | 9.696381028 | 1.17894404 | 0.121585985 | 2.264109984 | 0 |
| mmu-miR-138 | 8.988401863 | 0.58086385 | 0.064623707 | 1.495744597 | 0 |
| mmu-miR-181c | 8.702039479 | 0.50509257 | 0.05804301 | 1.419214415 | 0 |
| mmu-miR-382 | 7.650232283 | 0.48296538 | 0.063130812 | 1.397613433 | 0 |
| mmu-miR-19b | 7.113282862 | 0.85871061 | 0.120719312 | 1.81341687 | 0 |
| mmu-miR-381 | 6.596408588 | 0.57693327 | 0.087461724 | 1.49167503 | 3.550973654 |
| mmu-miR-666-3p | 4.508384346 | 1.18502318 | 0.262848749 | 2.273670484 | 5.326460481 |
| mmu-miR-376a | 4.506030766 | 0.77814194 | 0.172688999 | 1.714920793 | 5.326460481 |
| mmu-miR-873 | 4.382699353 | 0.58145566 | 0.13267067 | 1.496358294 | 5.326460481 |
| mmu-miR-181a | 4.327860643 | 0.34375539 | 0.079428479 | 1.269055695 | 5.326460481 |
| mmu-miR-383 | 4.321673066 | 0.54089814 | 0.125159431 | 1.45487796 | 5.326460481 |
| mmu-miR-181b | 4.231258159 | 0.3502464 | 0.082775947 | 1.27477833 | 5.326460481 |
| mmu-miR-99b | 4.127299977 | 0.51052094 | 0.123693684 | 1.424564495 | 5.326460481 |

| **Gene ID** | **Score** | **Numerator** | **Denominator** | **Fold Change** | **q-value (%)** |
| --- | --- | --- | --- | --- | --- |
| mmu-miR-1224 | -19.32225619 | -0.2257595 | 0.01168391 | 0.855144714 | 0 |
| mmu-miR-9* | -9.816858591 | -0.4221486 | 0.043002413 | 0.746312313 | 3.195876289 |
| mmu-miR-26a | -7.34181236 | -0.3084803 | 0.042016919 | 0.807491884 | 3.195876289 |
