# Supplementary material for: Integrated analysis of omics data using microRNA-target mRNA network and PPI network reveals regulation of Gnai1 function in the spinal cord of Ews/Ewsr1 KO mice
Source: BMC Med Genomics. 2016 Aug 12;9(Suppl 1):33. doi: 10.1186/s12920-016-0195-4 (PMC4989891; doi:10.1186/s12920-016-0195-4)
Supplement: Additional file 3: Table S2. — The number of genes targeted by each miRNAs by using TargetScan and miRDB. Prediction results by TargetScan and miRDB do not agree much. Union of target genes were further analyzed by performing. (DOCX 15 kb) [file 12920_2016_195_MOESM3_ESM.docx]

| **Up-regulated miRNA** | **number of predicted gene** | | |
| --- | --- | --- | --- |
|  | **TargetScan only** | **common** | **miRDB only** |
| mmu-miR-127 | 12 | 1 | 11 |
| mmu-miR-410 | 385 | 77 | 423 |
| mmu-miR-433 | 135 | 73 | 192 |
| mmu-miR-138 | 330 | 82 | 120 |
| mmu-miR-181c | 414 | 409 | 466 |
| mmu-miR-382 | 78 | 35 | 276 |
| mmu-miR-19b | 486 | 353 | 146 |
| mmu-miR-381 | 471 | 155 | 351 |
| mmu-miR-666-3p | 190 | 54 | 197 |
| mmu-miR-376a | 2 | 0 | 24 |
| mmu-miR-873 | 135 | 38 | 285 |
| mmu-miR-181a | 420 | 403 | 448 |
| mmu-miR-383 | 78 | 35 | 276 |
| mmu-miR-181b | 419 | 404 | 459 |
| mmu-miR-99b | 30 | 6 | 12 |
| **Down-regulated miRNA** | | | |
| mmu-miR-1224 | 68 | 8 | 195 |
| mmu-miR-9-3p | 0 | 0 | 433 |
| mmu-miR-26a | 408 | 279 | 243 |
