# Supplementary figures and images for: Integrated analysis of omics data using microRNA-target mRNA network and PPI network reveals regulation of Gnai1 function in the spinal cord of Ews/Ewsr1 KO mice
Source: BMC Med Genomics. 2016 Aug 12;9(Suppl 1):33. doi: 10.1186/s12920-016-0195-4 (PMC4989891; doi:10.1186/s12920-016-0195-4)

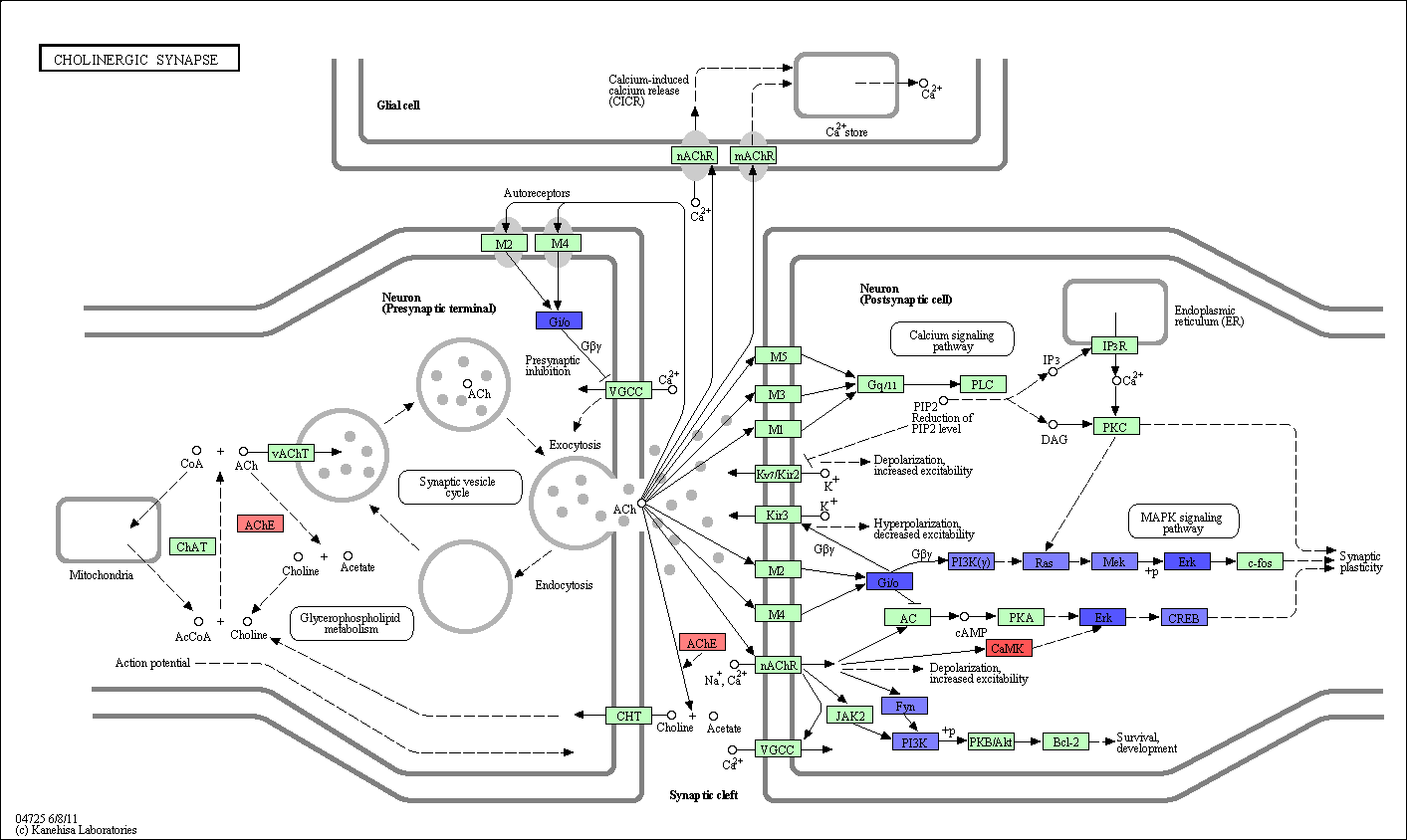

Supplement: Additional file 4: Figure S2. — The cholinergic synapse pathway related with significantly down-regulated genes by ClueGO. Selected DEGs are highlighted in colors chosen by KEGG mapper. Blue genes are down-regulated genes, and red genes are up-regulated genes in Ews/Ewsr1 KO mice compared to WT mice. Green color genes are not changed. (DOCX 53 kb) [file 12920_2016_195_MOESM4_ESM.docx]
